# Quantification of Cooperativity in Heterodimer-DNA Binding Improves the Accuracy of Binding Specificity Models*[Fn FN2]

**DOI:** 10.1074/jbc.M115.691154

**Published:** 2016-02-24

**Authors:** Alina Isakova, Yves Berset, Vassily Hatzimanikatis, Bart Deplancke

**Affiliations:** From the ‡ Institute of Bioengineering,; § Institute of Chemical Sciences and Engineering, Ecole Polytechnique Fédérale de Lausanne, and; ¶ Swiss Institute of Bioinformatics (SIB), CH-1015 Lausanne, Switzerland

**Keywords:** computational biology, cooperativity, DNA-binding protein, transcription, transcription regulation, heterodimer-DNA interactions, specificity models

## Abstract

Many transcription factors (TFs) have the ability to cooperate on DNA elements as heterodimers. Despite the significance of TF heterodimerization for gene regulation, a quantitative understanding of cooperativity between various TF dimer partners and its impact on heterodimer DNA binding specificity models is still lacking. Here, we used a novel integrative approach, combining microfluidics-steered measurements of dimer-DNA assembly with mechanistic modeling of the implicated protein-protein-DNA interactions to quantitatively interrogate the cooperative DNA binding behavior of the adipogenic peroxisome proliferator-activated receptor γ (PPARγ):retinoid X receptor α (RXRα) heterodimer. Using the high throughput MITOMI (mechanically induced trapping of molecular interactions) platform, we derived equilibrium DNA binding data for PPARγ, RXRα, as well as the PPARγ:RXRα heterodimer to more than 300 target DNA sites and variants thereof. We then quantified cooperativity underlying heterodimer-DNA binding and derived an integrative heterodimer DNA binding constant. Using this cooperativity-inclusive constant, we were able to build a heterodimer-DNA binding specificity model that has superior predictive power than the one based on a regular one-site equilibrium. Our data further revealed that individual nucleotide substitutions within the target site affect the extent of cooperativity in PPARγ:RXRα-DNA binding. Our study therefore emphasizes the importance of assessing cooperativity when generating DNA binding specificity models for heterodimers.

## Introduction

Mapping the interactions between transcription factors (TFs)^4^ and their DNA target sites is essential for elucidating the structural properties of gene regulatory networks (1, 2). Data on TF DNA binding specificities have so far revealed that individual TFs can bind to a broad set of target sites that cover a wide affinity range (3–6). In addition, it is now well appreciated that the binding of many TFs is not autonomous but is in fact influenced by a multitude of factors, including chromatin state, post-translational modifications, and interactions with other proteins. One specific form of protein interaction involves two TFs forming one heterodimeric DNA binding complex. Such heterodimers are highly abundant across organisms and exert essential molecular functions (2, 7, 8). Consequently, a lot of effort has been invested to determine their DNA binding specificities using various *in vitro* and *in vivo* approaches (7, 9–15). Several studies demonstrated the ability of two TFs to cooperate on DNA elements and thus provide an alternative mode of DNA recognition (16, 17). For example, Hox proteins gain novel specificities when bound to DNA together with the dimeric cofactor Exd (18). Sox-Oct partners, as well as certain nuclear receptor dimers, have different cooperativity constants when bound to DNA sites separated by spacers of variable length (17, 19, 20). But despite this clear demonstration of cooperativity phenomena, our ability to integrate its impact in quantitative models of DNA binding, and ultimately gene regulation, remains limited. Consequently, several important questions remain unaddressed. These include whether the perturbation of cooperative TF-DNA binding always involves major rearrangements of interacting molecules such as, for example, the addition or removal of a protein partner or introduction of a different spacer between two binding sites. In addition, it remains unclear whether cooperativity can be modulated on a much more fine-grained scale such as, for example, at the level of nucleotide variations in target binding sites. More specifically, it has not been comprehensively explored whether the information on the variable “strength” of cooperative effects in dimer binding to sites of different nucleotide composition could be used to refine a quantitative specificity model for the TF pair. Several quantitative models of TF-DNA binding specificity have been developed (3, 11, 21, 22), but none of these include to our knowledge the cooperative determinant of specificity. This knowledge gap reflects in large part the challenging nature of retrieving quantitative DNA binding parameters underlying heterodimer-DNA binding.

**FIGURE 1.**
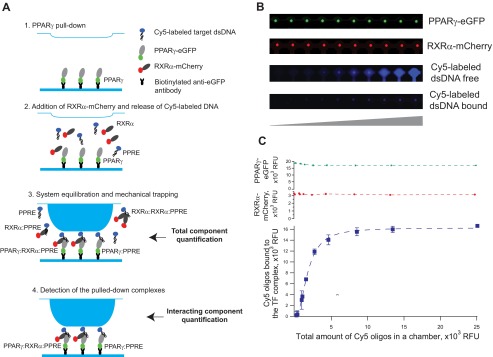
**On-chip heterodimer-DNA assembly.** *A,* schematic representation of the experimental setup. *Step 1,* PPARγ fused to an eGFP tag is immobilized on the surface of a MITOMI chip with an anti-GFP antibody. *Step 2,* RXRα tagged with mCherry and Cy5-labeled DNA baits are introduced into the system; *step 3,* immobilized PPARγ, RXRα, and DNA baits are then incubated for 1 h to allow system equilibration and complex assembly; *step 4,* newly formed complexes are trapped under a flexible PDMS membrane, and unbound molecules as well as molecular complexes are washed away. *B,* fluorescence-based readout of PPARγ-GFP, RXRα-mCherry, and Cy5-labeled target DNA from 10 MITOMI units. The *two upper panels* represent PPARγ-GFP and RXRα-mCherry detected in the center of each unit (under the PDMS membrane). The *two lower panels* represent the variable amounts of Cy5-labeled target DNA molecules detected in the same 10 MITOMI units, before (DNA-free) and after (DNA-bound) trapping. *C,* corresponding quantitative readout of *B* where the quantified amounts of both PPARγ and RXRα remain constant, but the amount of bound DNA increases with the input DNA concentration until it reaches saturation. The corresponding quantities of proteins and DNA are expressed in relative fluorescent units (*RFU*).

In this study, we addressed this challenge by using a robust microfluidics approach, MITOMI (23), that allows us to track and characterize the implicated molecular interactions in great quantitative detail. As a model system, we focused on the PPARγ:RXRα heterodimer. PPARγ is well known as one of the major regulators of adipocyte differentiation (24, 25), forming a DNA binding partnership with another nuclear receptor, RXRα, to control the adipogenic gene expression program. Generating a quantitative understanding of the molecular rules underlying the assembly of this heterodimer on DNA is therefore of gene regulatory as well as biomedical relevance. To accommodate the quantitative analysis of PPARγ:RXRα-DNA interactions, we expanded the previously described MITOMI setup by introducing and testing the usage of multiple fluorescent fusions with both heterodimer TFs, aiming to both track individual TFs as well as to monitor homo- and heterodimer formation on DNA (Fig. 1). We then used the MITOMI-derived data to assess the ability of the PPARγ:RXRα heterodimer to change its specificity upon dimerization as well as to support the development of a detailed quantitative binding model, specifically assessing the contribution of cooperativity to the DNA binding process. Using a comprehensive mechanistic modeling approach, we were able to derive affinity constants that account for cooperative heterodimer-DNA binding, allowing us to build a PPARγ:RXRα-DNA binding specificity model of greater predictive power than the one based on a regular one-site equilibrium. As such, our results provide unprecedented insights into the quantitative aspects of PPARγ:RXRα-DNA complex formation, emphasizing the role of binding site composition in influencing the cooperative nature of heterodimeric DNA binding.

## Experimental Procedures

### 

#### 

##### Device Fabrication

All the molds for microfluidic devices and devices itself were designed and fabricated as described previously (23, 26).

##### Synthesis and Printing of Target DNA Libraries

All target DNA fragments were obtained as single-stranded oligonucleotides from Invitrogen. These oligonucleotides were subsequently used to generate fluorescently labeled double-stranded oligonucleotides as described previously (23). The single base substitution libraries of PPRE, 5′-AAACTAGGTCAAAGGTCA-3′, and PAL3, 5′-AAACTAGGTCACCGTGACCT-3′, were generated by substituting one nucleotide of the elements at a time to all possible variants. All labeled double-stranded oligonucleotides were spotted onto epoxy-coated glass slides (CELL Associates) with a SpotBot III microarrayer (ArrayIT) using a 946MP4 pin (European Biotek Network SPRL).

##### Protein Cloning and Expression

TFs were expressed *in vitro* using the TnT SP6 High-Yield Wheat Germ protein expression system (Promega). To enable the expression of TFs and their fluorescence-based detection, we generated novel vectors by cutting the pF3A WG (BYDV) Flexi vector (Promega) with NcoI and DraI, removing the barnase cassette. The NcoI site was blunted, and the Gateway reading frame A cassette (Life Technologies, Inc.) was ligated. Subsequently, the eGFP and the mCherry coding sequence (EUROSCARF) containing a stop codon at the 3′-end were incorporated between the KpnI and SacI restriction sites using standard cloning techniques. Full-length PPARγ and RXRα ORFs were then subcloned from the Entry clones (27) into the generated vectors by standard Gateway cloning.

##### MITOMI and Data Analysis

The surface chemistry, MITOMI, and image acquisition were performed as described previously (23). We quantified the amount of each mutated sequence bound to the respective TF at the equilibrium state by means of fluorescence in a range of input DNA concentrations. The obtained equilibrium binding curves for each sequence were then fitted with the regression curves generated from the proposed model of cooperative binding.

### Binding Model

#### 

##### Monomer-DNA Interactions

In the case of a single TF-DNA interaction at equilibrium,

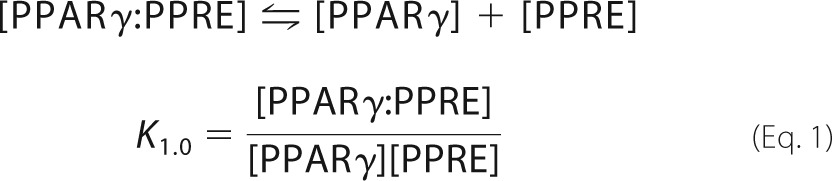


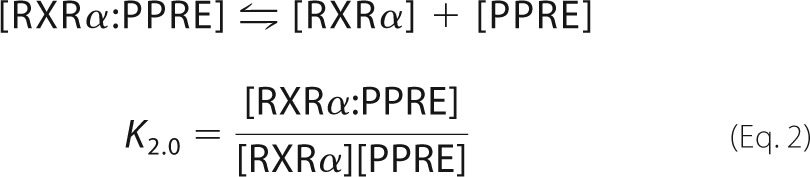
 where *K*_1,0_ and *K*_2,0_ are the respective PPARγ- or RXRα-DNA binding constants that are mutation-dependent (see supplemental material). For monomer-DNA interactions, the binding curves were fitted with a single-parameter non-linear function. For each sequence, the fit that yielded the lowest χ^2^ value was used to compute the function parameter (binding constant). The accuracy of the fitting parameters was assessed via residuals of the fit. The standard deviation (σ) of the binding constant was computed for each sequence (supplemental Table S1).

##### Heterodimer-DNA Interactions

In the case of heterodimer-DNA interactions, we accounted for the number of all possible molecular species that could be formed between all three components. We formed a system of two different sites and two ligands, similar to Ref. 28, with the following additional properties: we allowed RXRα to dimerize with itself or with PPARγ, and we allocated two binding sites for RXRα (left and right, equal binding affinity), with one of them (left) also able to bind PPARγ. These considerations led to the definition of the following species: PPRE (*X*_0_); PPARγ (*X*_1_); RXRα (*X*_2_); PPARγ:RXRα (*X_D_*_1_); RXRα:RXRα (*X_D_*_2_); PPARγ:PPRE (*X*_10_); RXRα:PPRE (*X*_20_); PPRE:RXRα (*X*_02_); PPARγ:PPRE:RXRα (*X*_12_); RXRα:PPRE:RXRα (*X*_22_); PPARγ:RXRα:PPRE (*X*_120_); PPRE:RXRα:PPARγ (*X*_012_); PPRE:RXRα:RXRα (*X*_022_); RXRα:RXRα:PPRE (*X*_220_); and RXRα:PPARγ:PPRE (*X*_210_); where the notation PPARγ:PPRE:RXRα (*X*_12_) indicates that PPARγ binds to the left binding site of PPRE and RXRα to the right one. PPARγ:RXRα:PPRE (*X*_120_) indicates that the PPARγ:RXRα heterodimer binds PPRE only via RXRα.

**SCHEME 1.**
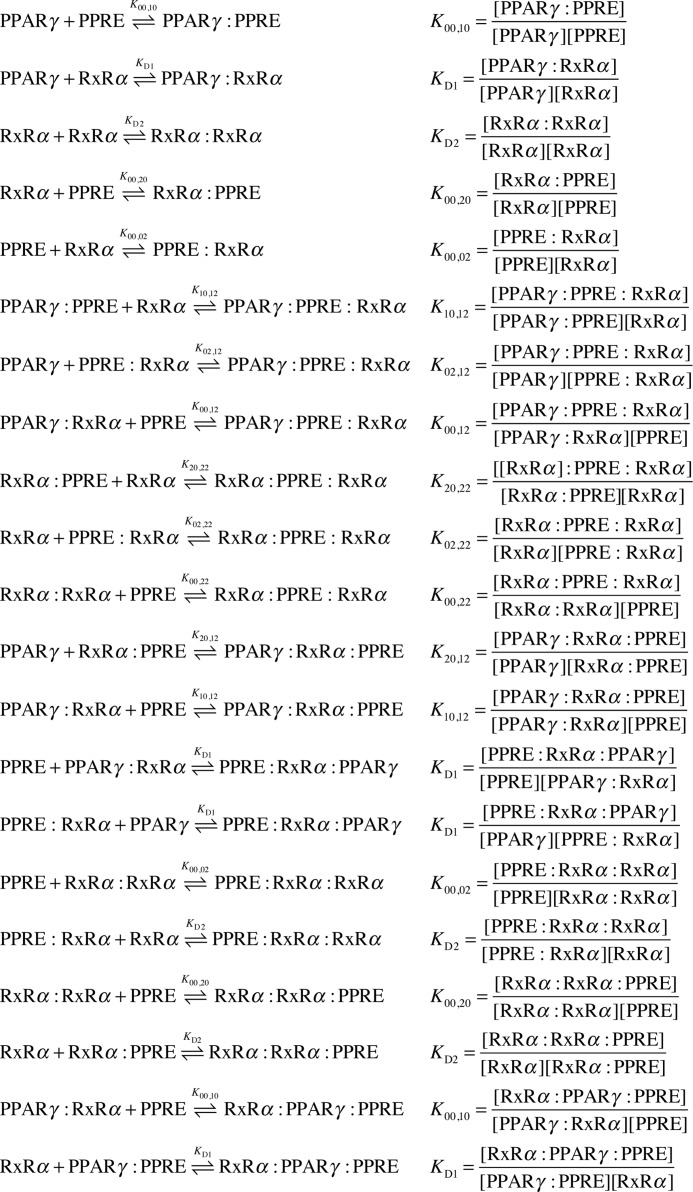


All possible elementary interactions between PPARγ, RXRα, and PPRE are shown in Scheme 1. From the above relations, we define *K*_DoD_ as the product of the binding affinities involved in each of the possible heterodimer on DNA formation pathways that we denote Equation 3,

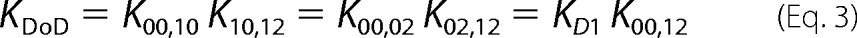
 After the assignment of experimental values to *K*_00,10_, *K*_00,02_, *K_D_*_1_, and *K_D_*_2_ measured in a previous experiment, the system remains with two independent parameters, *K*_00,12_ and *K*_00,22_. We solve the system at equilibrium, *i.e.* find the species concentrations such that all equilibrium relations are fulfilled. We calculate the fraction of PPRE involved in complexes with PPARγ and find the parameters *K*_00,12_ and *K*_00,22_ such that the simulation best fits the experimental measurements of PPRE bound to immobilized PPARγ in the least squares sense. The accuracy of each fit was assessed through the residual sum of squares value (see RSS, supplemental Table S1). The binding parameters were calculated from the best fits. The simulations were performed with Matlab (Mathworks).

##### Cooperativity

We next use the values of the ternary complexes *K*_00,12_ and *K*_00,22_ derived from the model fits to assess the presence or absence of cooperative effects in heterodimer-DNA binding. Cooperativity effects can be quantified at the steady-state through the cooperativity factors shown in Equation 4,

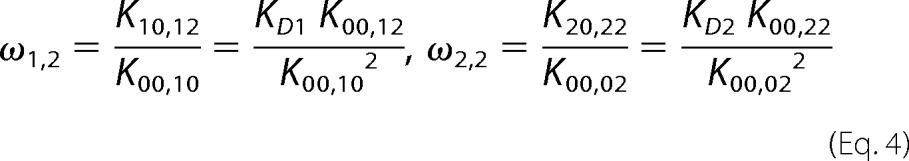
 where ω_1,2_ and ω_2,2_ are defined strictly as the ω coefficient presented in Ref. 17. The cooperativity factors can take any value greater than 0; cooperativity is positive when ω > 1 and negative when ω < 1. Note that this formulation quantifies the effect of cooperativity but does not elucidate its molecular nature, *i.e.* cooperativity can be due to direct ligand-ligand interactions or indirect communication between the ligands (29). The goodness-of-fit of all the simulations were measured via the residuals of the unweighted least squares (supplemental material).

##### Motif Enrichment in ChIP-seq Data

ChIP-seq-based PPARγ:RXRα DNA binding regions in 3T3-L1 cells were retrieved from Nielsen *et al.* (30) and processed as in Raghav *et al.* (31). Area under the receiver operating characteristic curve (AUC) representing the binding site occupancy predicted by the binding model was calculated as described previously (32) in that a 200-bp region around the center of the peak was used as the positive binding region and a 200-bp-long genomic sequence 300 bp downstream of the peak center as the negative binding region. Three motifs were used in the search as follows: 1) PSSM motif derived from *K_d_* values; 2) PSSM motif derived from *K*_DoD_ values; 3) JASPAR motif (MA0065.2, JASPAR CORE Database).

## Results

### 

#### 

##### Benchmarking of MITOMI-based PPARγ:RXRα-DNA Interaction Analyses

Recent ChIP-seq (30), ChIP-chip (33), and ChIP-PET (34) analyses revealed that the PPRE is the main *cis*-acting element for high affinity tethering of PPARγ:RXRα heterodimers to the DNA. The PPRE contains two copies of the 5′-AGGTCA-3′ consensus half-site separated by one nucleotide, constituting the so-called DR1 element, as well as a 5′-AAACT sequence that has been shown to be important for PPRE recognition by PPARγ (35). To benchmark our MITOMI approach, we first investigated the ability of *in vitro* expressed PPARγ, RXRα, and the heterodimer PPARγ:RXRα to preferentially bind to PPRE, as compared with other previously characterized nuclear receptor-binding sites such as the estrogen- and glucocorticoid-response elements, canonical AGGTCA repeats separated by one or three nucleotides (DR1 and DR2 sites) and variants thereof, as well as the PAL3 element and variants thereof.

**FIGURE 2.**
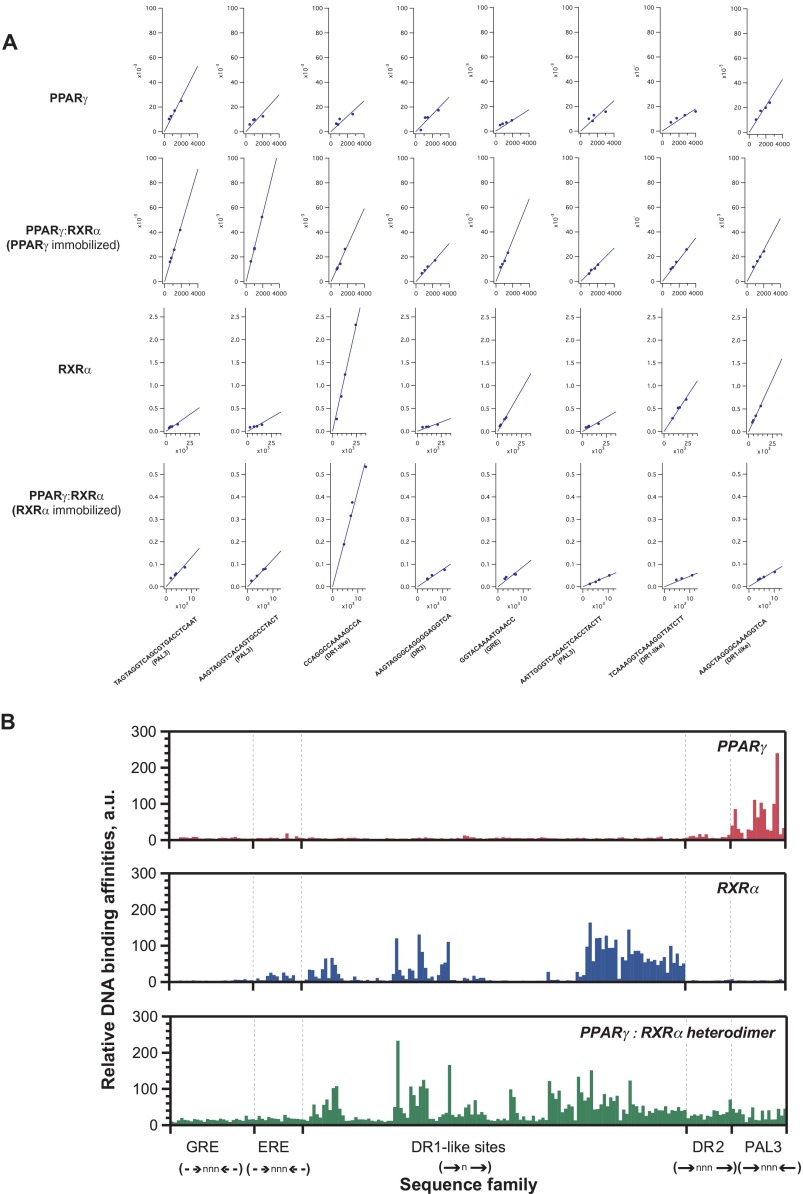
**DNA binding preferences of PPARγ, RXRα, as well as the PPARγ:RXRα heterodimer.** *A,* linear fits of binding data. Examples of binding curves and corresponding linear fits of PPARγ, RXRα, and PPARγ:RXRα heterodimer interactions with sequences containing putative nuclear receptor binding sites. *B,* relative DNA binding affinities of PPARγ, RXRα, and the PPARγ:RXRα heterodimer to five putative nuclear receptor-binding sites and variants thereof. Each sequence family is defined by the orientation of the canonical hexameric sites (represented by *arrows*) and the spacing between them.

Because of the scalability of the MITOMI chips compared with traditional methods such as the gel shift assay, we were able to screen the entire library consisting of 192 sequences at four different DNA concentrations, against either PPARγ or RXRα alone or the PPARγ:RXRα dimer in a single MITOMI experiment. This is important because it allowed us to directly compare the relative DNA affinity of a certain TF for each sequence at uniform surface preparation, conditions, and sample handling. To evaluate the DNA binding preferences of PPARγ, RXRα, and PPARγ:RXRα dimers within the queried nuclear receptor DNA binding site space, we quantified DNA bound to the TFs at the equilibrium state. For each sequence, we plotted the amounts of DNA bound by the TF and normalized by protein levels *versus* the total input DNA at four different concentrations, which all fell below half the binding saturation level (*i.e.* in a linear range of the binding curve) (Fig. 2*A*). We then estimated the relative DNA affinity of PPARγ, RXRα, and the heterodimer to given sequences as slopes of linear regression curves fitted to the data points (Fig. 2*B*).

**FIGURE 3.**
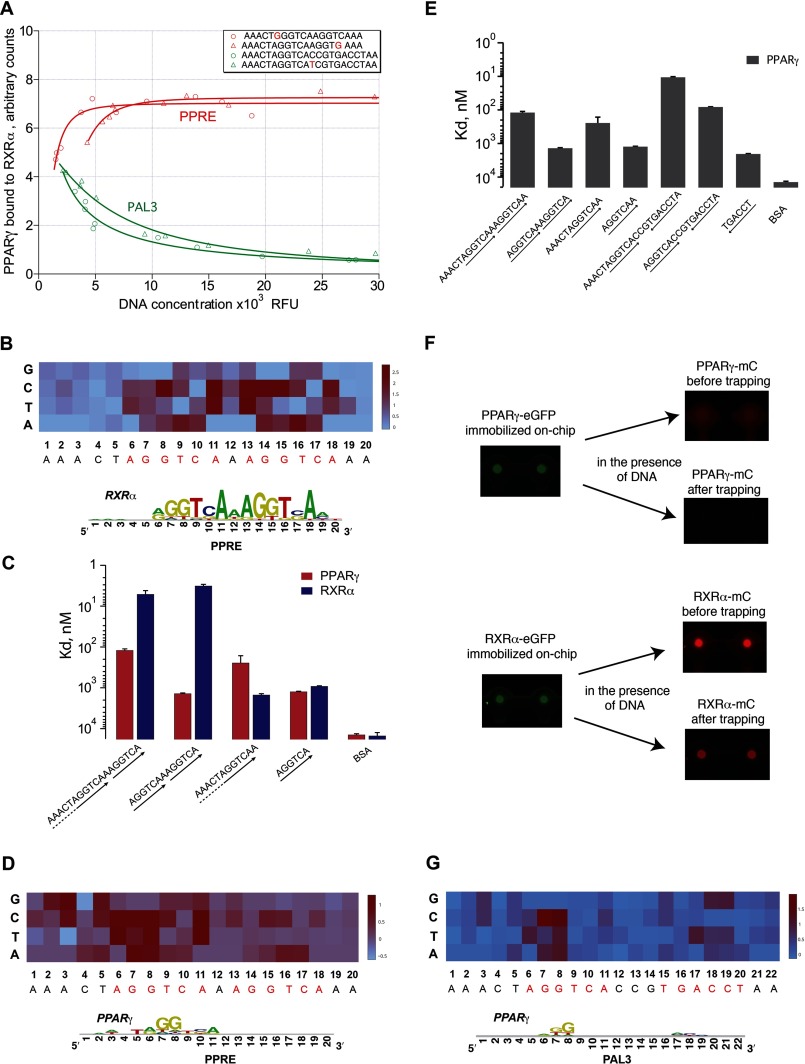
**DNA binding behavior of PPARγ and RXRα on PPRE, PAL3, or variants thereof.** *A,* heterodimer formation in the presence of PPRE and PAL3 DNA at different concentrations. *B,* DNA binding landscape of RXRα monomer to single nucleotide variants of PPRE. The heatmap represents the mean of ddG values (the difference in Gibbs energy of RXRα binding to a mutant site compared with the energy of RXRα binding to canonical PPRE) derived from two independent MITOMI experiments. The sequence of the canonical PPRE is indicated along the *x* axis. Two core hexamer repeats, constituting the DR1, are highlighted in *red. Below heatmap*: energy-normalized sequence logo (39) derived from the matrix of the binding energy contribution for each base at each position of PPRE. *C,* binding affinities of PPARγ or RXRα to DR1 and PAL3 sites or truncated variants thereof. *D,* same as for *B*, but for PPARγ instead of RXRα. *E,* binding affinities of PPARγ to variants of DR1 and PAL3 sites. *F,* visualization of on-chip assembly of putative PPARγ and RXRα dimers. *mC* refers to the fluorescent tag mCherry (*red*). *G,* DNA binding landscape of PPARγ monomer to PAL3 or single nucleotide variants thereof. Each *bar* represents the mean and standard deviation of ddG derived from two independent MITOMI experiments. *Below heatmap*: energy normalized sequence logo (39) derived from the matrix of the binding energy contribution for each base at each position in the PAL3 element.

We found the binding preferences of PPARγ, RXRα, or PPARγ:RXRα heterodimer detected within our MITOMI assay (Fig. 2) to be consistent with previously identified DNA binding specificities for these TFs, both *in vitro* and *in vivo* (30, 36), thus validating our approach. For example, we observed that the affinity of RXRα to DR1-like sites is significantly greater than to glucocorticoid- or estrogen-response element-like elements. In contrast, we found that PPARγ weakly binds to direct repeat sites but strongly to the PAL3 element, as reported previously (41, 44). However, in the presence of RXRα, PPARγ shifted its specificity to DR1-like sites and no longer exhibited a preference for the PAL3 element. We confirmed this observation by performing independent MITOMI experiments in which we measured the amount of PPARγ that is interacting with RXRα in the presence of either PPRE or PAL3 sites (Fig. 3*A*). We fixed the amount of RXRα molecules by immobilizing them on the surface of the chip and introduced PPARγ in amounts that were sufficient to saturate the binding to RXRα while varying the amount of accessible DNA. When using low DNA concentrations, the amount of formed heterodimer was similar for both PPRE and PAL3 elements. However, upon increasing the amount of PPRE target DNA, we observed an increase in heterodimer formation. In the presence of PAL3, we observed the opposite effect as the amount of formed heterodimer decreased, suggesting that PPARγ was bound by PAL3 and thus sequestered from the TF partner (Fig. 3*A*). Together, our results clearly demonstrate that also in our MITOMI assay, PPRE is the site to which PPARγ:RXRα has the highest affinity. We therefore decided to use this site for an in-depth TF-TF-DNA binding characterization.

##### PPARγ and RXRα Exhibit Intrinsic Affinity to the PPRE Prior to Dimerization

We performed a detailed analysis of monomeric RXRα and PPARγ binding to the PPRE (Fig. 3, *B* and *D*). To investigate the contribution of each nucleotide within the PPRE to the binding specificity of each tested monomeric TF, we generated a single base substitution library of PPRE whereby we substituted each base pair of the element, one nucleotide at a time. We then quantified the TF-bound amount of each mutated sequence at the equilibrium state in a range of input DNA concentrations. We fitted obtained binding curves with the model streamlined for monomeric TF-DNA binding (model fits and corresponding residuals are demonstrated in supplemental material). Next, we derived the equilibrium binding constants of PPARγ-PPRE and RXRα-PPRE interactions after which we calculated the differences in binding energy between each sequence of the library and the canonical, non-mutated PPRE (Fig. 3, *B* and *D*). Using these values, we subsequently derived the position-specific scoring matrix for PPARγ and RXRα binding to the PPRE and plotted corresponding enoLOGOS (Fig. 3, *B* and *D*) (39). This approach has been shown to accurately describe the DNA binding specificities of TFs, even though it assumes that each nucleotide of the element contributes to TF binding independently (23, 40). We found the following: 1) RXRα binding to PPRE is highly specific such that even a single nucleotide substitution within the core DR1 motif causes a significant change in binding energy (Fig. 3*B*); 2) the 5′-AGGTCA-3′ is the energetically favorable hexameric motif for RXRα monomer binding (Fig. 3, *B* and *C*) consistent with results from previous studies (41–44); 3) due to the symmetry of the DR1 element, RXRα can bind to either of the two hexameric half-sites (Fig. 3, *B* and *C*); and 4) the binding energy does not change significantly upon the addition of flanking bases up- or downstream of the AGGTCA sequence indicating that 6 bp are sufficient to accommodate an RXRα molecule (Fig. 3*C*).

Interestingly, we observed that PPARγ, even without an RXRα partner, shows sequence-specific binding to PPRE, with its target site located near the 5′-end of the element (Fig. 3*D*). Unlike RXRα, sequence-specific DNA binding of PPARγ was not restricted to the 5′-AGGTCA-3′ half-site. The DNA binding energy of PPARγ also changed upon the substitution of bases that are located upstream of this core site, and the 5′-AACT element of the DR1 half-site is required for a specific interaction (Fig. 3, *D* and *E*). This observation supports the importance of this upstream element in mediating the stabilization of the C-terminal extension of the DNA binding domain of PPARγ, as reported previously (45).

##### PPARγ Binds to PAL3 with High Affinity in the Absence of RXRα

Consistent with earlier reports (41, 44), we found that PPARγ binds to the PAL3 element (Fig. 2*B*). It was suggested however that this involves PPARγ homodimerization. To test the ability of PPARγ to form a homodimer in solution as well as on DNA, we first expressed PPARγ with an eGFP fusion and immobilized it on the surface of the chip. After extensive washing of the surface aiming to disrupt putative PPARγ-eGFP dimers, we introduced PPARγ fused to mCherry to the device releasing the Cy5-labeled PAL3 element at the same time. Our analyses showed strong binding of PPARγ to the PAL3 element, yet no homodimerization of PPARγ was observed because mCherry-derived fluorescence could not be detected. Thus, these data suggest that PPARγ binds to the PAL3 element as a monomer (Fig. 3*F*).

To investigate the DNA binding properties of PPARγ to the PAL3 element, we established the DNA binding landscape between this TF and respective target sequences in a fashion similar to our analyses of PPARγ and RXRα on PPRE (Fig. 3*G*). Interestingly, upon interacting with the PAL3 motif, we found that PPARγ tolerates a greater sequence degeneracy compared to when it is interacting with PPRE. This is reflected by the low information content of the sequence logo revealing the DNA binding specificity of PPARγ on PAL3 (Fig. 3*G*), and it could be due to the palindromic nature of the PAL3 element (Fig. 3*G*). In addition, we found that the affinity of PPARγ alone for the PAL3 element is greater than that for PPRE (Fig. 3*E*). This could be explained by the role of flanking bases located downstream of the canonical AAACTAGGTCA site that may stabilize a PPARγ molecule on DNA. To test this hypothesis, we measured the binding affinities of PPARγ to PAL3 sequence variants in which we systematically removed 1 bp starting from the 5′- or 3′-ends (data not shown). We generally observed an affinity decrease when a sequence shorter than AAACTAGGTCACCGTGA was screened with PPARγ. Thus, our data support a model in which PPARγ binds in monomeric fashion to the PAL3 element, which is favored over the DR1 element because of the presence of additional bases downstream of the canonical 5′-AGGTCA-3′ repeat.

##### PPARγ and RXRα Bind PPRE in a Cooperative Fashion

To characterize the biophysical properties of PPARγ:RXRα binding to DNA, we implemented a similar approach as the one used for characterizing monomeric TF DNA binding. We measured the DNA occupancies of PPARγ:RXRα on each sequence belonging to the PPRE single base substitution library and derived equilibrium binding curves of the heterodimer with respect to different variants of the PPRE. However, a putatively confounding factor that may skew the quantification of heterodimer-bound DNA is the ability of RXRα to bind DNA as a homodimer (44) that can compete with the heterodimer PPARγ:RXRα for binding to PPRE (Fig. 1*A*, *step 3*). To eliminate or at least reduce this bias, we opted to perform DNA binding experiments in which GFP-tagged PPARγ and not RXRα is immobilized on the surface of the chip such that mCherry-tagged RXRα is present at the “detection” area under the MITOMI button only when bound to PPARγ (Fig. 1*A*). Nevertheless, we measured PPARγ:RXRα DNA binding in the two configurations (in which either PPARγ or RXRα is immobilized on chip) and obtained highly correlated relative affinity values (*R*^2^ = 0.84) for heterodimer binding to each PPRE mutant, suggesting that the order bias may not be as large as initially hypothesized.

**FIGURE 4.**
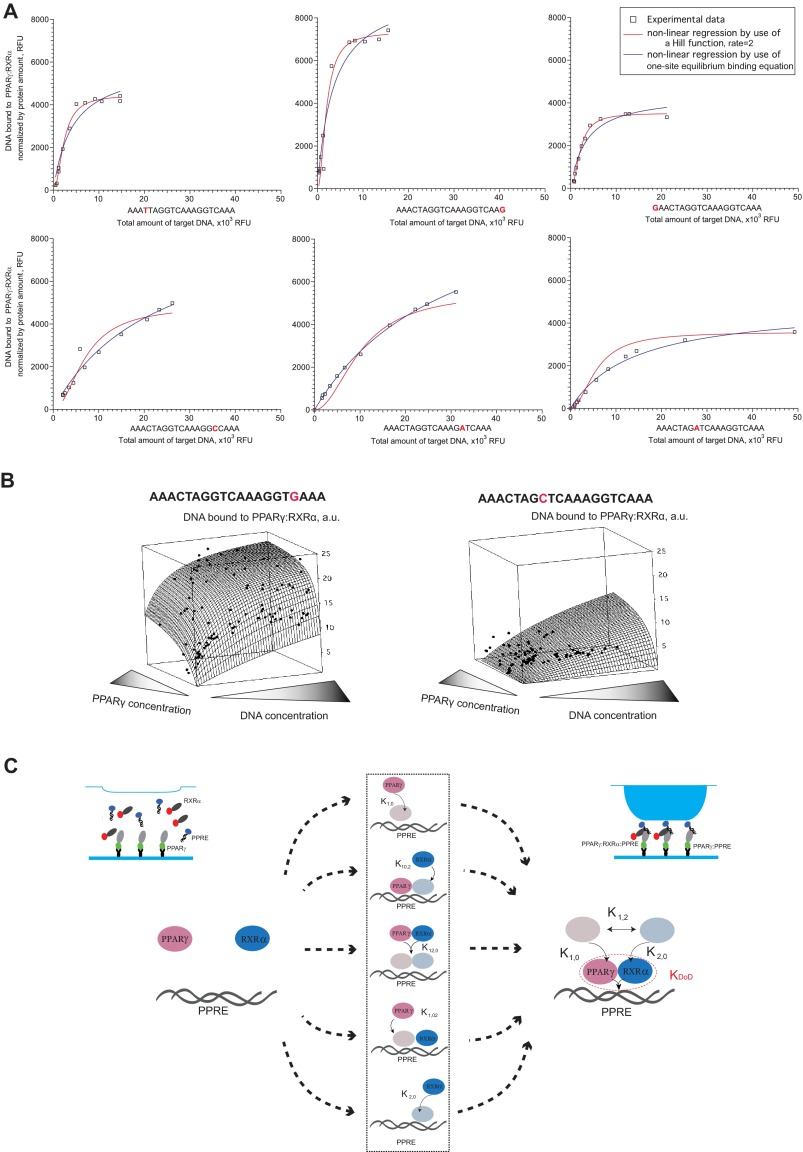
**Cooperative TF-DNA interactions.** *A,* examples of binding curves representing PPARγ:RXRα binding to PPRE or variants thereof. The nucleotide that was substituted in each sampled sequence is highlighted in *red. B,* binding of the PPARγ:RXRα heterodimer to the DR1 element in function of different DNA and PPARγ concentrations. One example of a strongly (*left*) and weakly (*right*) bound sequence, respectively, is shown. Raw experimental data are represented by *black dots,* and the surface plot represents the regression of the data using Voronoi interpolation. The amount of bound DNA is expressed in arbitrary units (*a.u.*). *C,* schematic representation of various scenarios of heterodimer formation. We allow the heterodimer to be formed through either the monomer or dimer scenarios.

We first applied simple one-site equilibrium models for DNA binding (23, 46) to describe the heterodimer-DNA interactions, but these models failed to explain the MITOMI binding data of the PPARγ:RXRα heterodimer to PPRE and variants thereof (Fig. 4*A*). Specifically, the experimental binding curves exhibited distinct behavioral modes depending on the composition of the DNA target site. The majority of the binding curves exhibited sigmoidal behavior suggesting that PPARγ and RXRα bind DNA in a cooperative manner (Fig. 4*A*). Interestingly, certain substitutions within the AGGTCA repeat significantly affected the shape of the binding curves. For example, upon substitution of the guanines in the AGGTCA core, the DNA binding curve of the dimer did not display a sigmoidal behavior; rather, it followed the shape of a hyperbolic function that typically characterizes one-site binding curves (Fig. 4*A*).

Next, we asked how much the DNA binding behavior of the heterodimer depends on the abundance of PPARγ given that we previously showed that RXRα is 4–5-fold more abundant than PPARγ in terms of nuclear protein copies in adipocytes (47). To address this question, we analyzed binding of PPARγ:RXRα to several PPREs in the presence of different DNA and protein concentrations. We then represented the data obtained for each sequence as a three-dimensional scatterplot in which the DNA and PPARγ concentrations were projected onto the *x* and *y* axis, respectively, and the amount of DNA bound to an immobilized heterodimer on the *z* axis (Fig. 4*B*). We observed that the DNA binding occupancy of the heterodimer depends both on the DNA concentration and on the concentration of PPARγ. Collectively, these observations led us to hypothesize that DNA binding of the PPARγ:RXRα heterodimer is achieved through a complex cooperative mechanism clarifying why standard equilibrium binding models may be inadequate to define the binding parameters of PPARγ:RXRα-DNA interactions.

##### Mechanistic Model of Cooperative PPARγ:RXRα DNA Binding

We next asked whether the DNA binding behavior of the heterodimer could be explained by a single model of PPARγ:RXRα DNA binding based only on the knowledge of binding constants between each of the binding partners and PPRE. To address this question, we used the mass action reversible forms that were previously shown to mechanistically explain the binding of regulatory proteins to DNA (48). As a first step, we described all the elementary reactions in the PPARγ:RXRα-PPRE binding process and generated the mass balance equations that describe the formation of the binding species (Fig. 4*C*). Then, we used the knowledge on the energies of TF binding to DNA as single units as well as the energy of TF-TF interactions from the independent experiments introduced above to define corresponding parameters of the model. Solving the obtained mass balance equations for equilibrium binding, we estimated the affinity constants of ternary complexes to each PPRE mutant based on the best model fits to our data (Fig. 4*C* and supplemental material).

**FIGURE 5.**
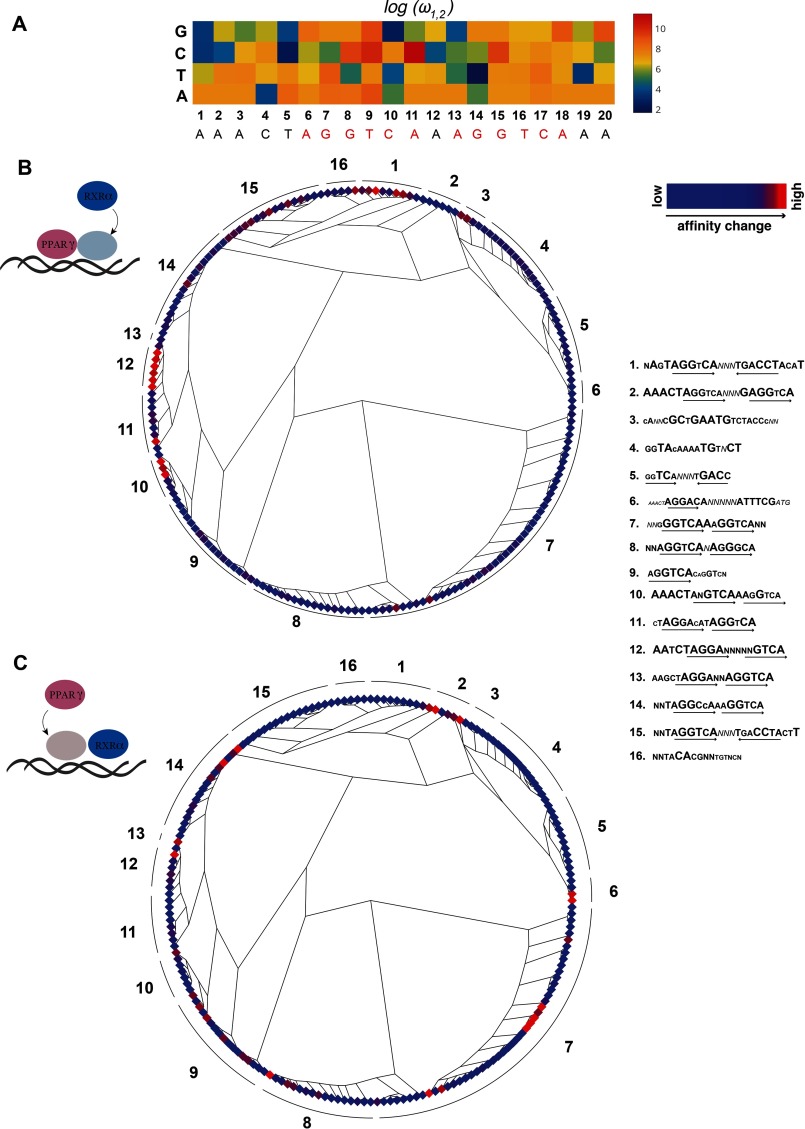
**Significance of cooperative effects in PPARγ:RXRα-DNA binding.** *A,* cooperativity map represents log ω_1,2_ values calculated for each PPRE variant. *B,* DNA affinity change (σ) upon PPARγ heterodimerization with RXRα. 192 sequences were clustered using MAFFT and plotted as a phylotree. The representative sequence of each subtree is denoted outside of the *tree circle*. The values of occupancy change observed for each sequence are plotted as color plots at the terminal nodes of the phylotree. *C,* same as *B*, but for RXRα heterodimerization with PPARγ.

To determine the significance of cooperative effects in PPARγ:RXRα-PPRE binding, we quantified the cooperativity factor ω (17) of PPARγ:RXRα binding to each PPRE variant, which allowed us to profile the whole spectrum of cooperativity constant values within the PPRE mutant library (Fig. 5*A* and supplemental material). We found that ω is much greater than 1 (ω ≫1) for all tested sequences (Fig. 5*A* and supplemental material). We also observed that single nucleotide changes within the PPRE do not equally affect the ability of the heterodimer to cooperate on the respective site. Specifically, we found that nucleotide changes in the first AGGTCA half-site tend to have a greater impact on ω (*i.e.* for the majority of nucleotide substitutions at PPRE positions 1–11, the value of ω_1,2_ varies more than for changes in the second half-site) (Fig. 5*A*). As indicated above, this upstream PPRE region is bound by PPARγ through DNA binding domain-DNA contacts that are additionally stabilized by the interaction of a hinge region of the protein with a minor groove at the 5′-end of PPRE (45). Thus, PPARγ does not only contribute to the specificity of the heterodimer, but our data indicate that it may also modulate the extent of cooperativity with RXRα on its target DNA sequence.

To investigate whether this cooperativity effect could also be observed when the heterodimer is bound to sites other than PPRE, we revisited our MITOMI data for 192 sequences representing various nuclear receptor response elements. However, for this DNA library, we were not able to directly quantify ω as we only measured relative affinities and did not generate the type of comprehensive binding data that we acquired for our single nucleotide substitution library. To resolve this issue, we estimated ω using the proxy value σ (with σ ∼ ω), which we defined here as the affinity change upon the addition of heterodimer partner for both PPARγ and RXRα as follows: σ_PPARγ-PPARγ:RXRα_ = affinity_**PPARγ**:RXRα_/affinity_RXRα_; and σ_RXRα-PPARγ:RXRα_ = affinity_PPARγ:**RXRα**_/affinity_PPARγ_, with the TF listed in bold being the one that was tethered to the surface of the MITOMI device.

We investigated the change of σ between different types of binding sites. Because estrogen- and glucocorticoid-response elements and PAL3 are essentially all palindromes separated by one nucleotide and some DR1 sequences are more similar to one another than to others, we first identified the similarity pattern between all 192 sequences. We independently aligned all sequences using MAFFT clustering (49), identifying 16 distinct target sequence clusters, and we plotted the σ values for each of the sequences contained within each cluster (Fig. 5, *B* and *C*). As expected, we found that the distribution of σ values for the majority of sequences is consistent with the clustering pattern. Interestingly, however, we also observed that for some sequence-homologous sites, the affinity of PPARγ to DNA significantly changes upon the presence of RXRα, as exemplified by PPRE-like type binding sites such as AATCTAGGANNNNNGTCA (Fig. 5*B*). Similarly, we observed an RXRα affinity increase upon the presence of PPARγ for PPRE-like sites as well as for DR4-like sites (AAACTAGGTCANNNGAGGTCA) (Fig. 5*C*). In both of these cases, we found that the affinity change could be different, even between very similar sequences (Fig. 5, *B* and *C*, *i.e. red* and *blue diamonds* within the same sequence cluster). This result is in line with our observation described above in that not only the orientation and spacing between the half-sites appears to affect heterodimer-DNA binding cooperativity but also the nucleotide composition of the target sites themselves.

##### Apparent DNA Binding Affinity Constant of a Heterodimer

**FIGURE 6.**
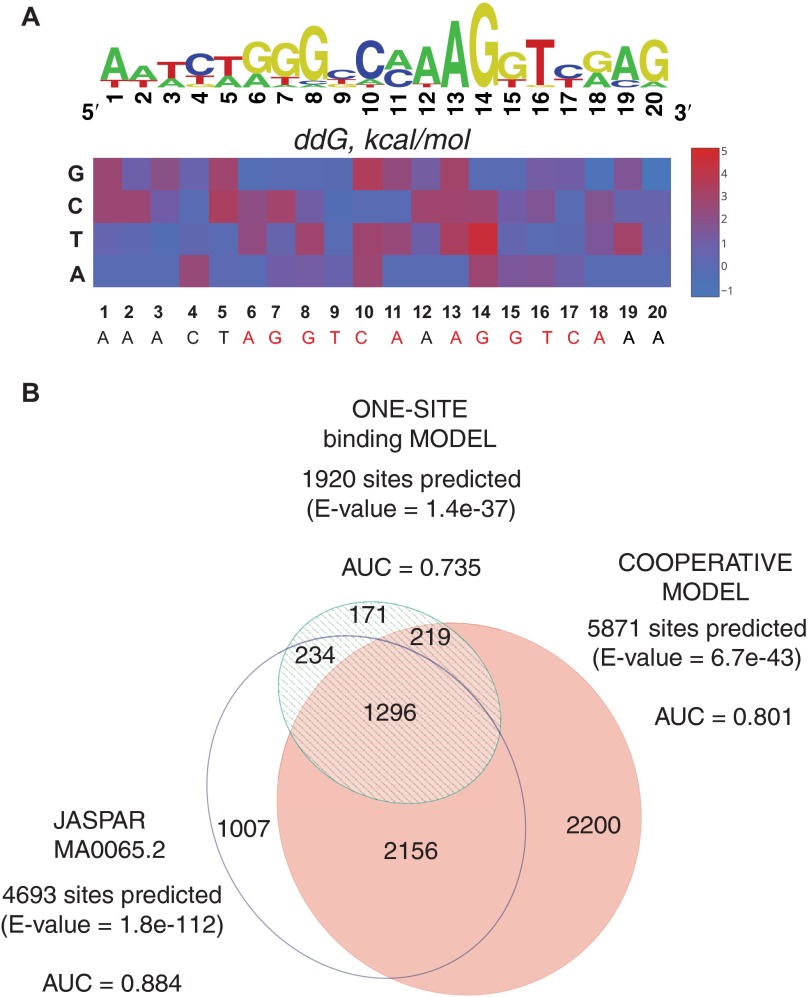
**Prediction of *in vivo* binding.** *A,* affinity map as well as the corresponding sequence logo (energy normalized sequence logo) (39) of PPARγ:RXRα heterodimer binding to PPRE. The affinity map represents the *K*_DoD_ values as calculated based on our cooperativity model. *B,* Venn diagram of the number of PPARγ:RXRα binding sites predicted by three different specificity models independently. The PPARγ:RXRα motif occurrence predicted within 200-bp genomic regions identified through ChIP-seq at day 6 of 3T3-L1 adipocyte differentiation.

The above results emphasize the important role of cooperativity in defining specific heterodimer-DNA binding. To investigate whether incorporating cooperativity into quantitative DNA binding models could enhance the quality of the model and thus improve our ability to predict *in vivo* heterodimer DNA binding, we quantified the cooperativity-inclusive parameters of PPARγ:RXRα-PPRE binding. We defined the affinity of the heterodimer to PPRE through the apparent DNA binding affinity constant of a heterodimer (*K*_DoD_) as the product of the binding affinities involved in each of the possible heterodimers on DNA formation pathways, and we estimated the *K*_DoD_ of PPARγ:RXRα for each single base pair substitution variant of PPRE from the experimental MITOMI data (Fig. 6*A* and supplemental material). We next decided to investigate whether the *K*_DoD_ reflects heterodimer-DNA binding more accurately than a canonical *K_d_*. To address this question, we fitted the experimental data with a one-site binding function, quantified corresponding *K_d_* values, and built a position-specific scoring matrix of PPARγ:RXRα binding to PPRE (Table 1). We then assessed how well either the cooperativity model-based motif (derived from *K*_DoD_ values) or the motif generated from the one-site binding model (derived from *K_d_* values) predicted *in vivo* PPARγ:RXRα binding in mature 3T3-L1 adipocytes (*i.e.* day 6 of adipogenesis, the time point of maximal PPARγ binding (30)), using as a reference the JASPAR motif that was derived from the PPARγ:RXRα ChIP-seq data itself. To do so, we computed the occurrence of either of the three motifs within previously published PPARγ:RXRα ChIP-seq data sets (30) and subsequently generated the area under a receiver operating characteristic (area under the receiver operating characteristic curve) scores for each motif (32). Our results showed that although the JASPAR motif scored best, as expected, our cooperativity model predicts PPARγ:RXRα *in vivo* DNA binding more accurately than the single-site model (area under the receiver operating characteristic curve of 0.801 compared with 0.731 for the one-site binding model-derived motif and 0.884 for the JASPAR motif) (Fig. 6*B*). In line with these results, we also found that the *K*_DoD_-based motif predicts a larger number of PPARγ:RXRα ChIP-seq peaks compared with the *K_d_*-based one: 5871 *versus* 1920 out of 10,114 total peaks (with the JASPAR motif predicting 4693 peaks). To confirm that the peaks predicted by our cooperativity model but not predicted by the JASPAR motif also contained the PPRE motif, we performed a MEME (Multiple Em for Motif Elicitation) (50) motif search on these peaks and identified the canonical AGGTCA repeat separated by one nucleotide as the main enriched motif (data not shown). Together, these results indicate that the accuracy of the specificity model of PPARγ:RXRα DNA binding increases when accounting for cooperativity effects in heterodimer-DNA binding.

**TABLE 1:** **PSSM matrices of PPARγ:RXRa** Alphabet = ACGT, strands: +, background letter frequencies (from uniform background): A 0.25 C 0.25 G 0.25 T 0.25.

**Motif *K_d_*_kcal/mol PPARγ::RXRα**			
0.110	0.411	0.360	0.120
0.127	0.183	0.183	0.507
0.429	0.120	0.322	0.130
0.144	0.196	0.423	0.237
0.165	0.186	0.359	0.290
0.137	0.149	0.189	0.525
0.226	0.326	0.206	0.242
0.365	0.199	0.233	0.202
0.421	0.208	0.166	0.205
0.404	0.160	0.277	0.159
0.195	0.224	0.367	0.214
0.192	0.293	0.324	0.191
0.194	0.171	0.302	0.333
0.221	0.304	0.276	0.198
0.264	0.228	0.228	0.279

**Motif *K*_DoD__kcal/mol PPARγ::RXRα**			
0.083	0.377	0.241	0.299
0.401	0.062	0.083	0.454
0.363	0.099	0.444	0.093
0.222	0.071	0.415	0.292
0.225	0.238	0.453	0.084
0.171	0.314	0.249	0.266
0.132	0.666	0.084	0.118
0.410	0.401	0.098	0.092
0.447	0.081	0.214	0.258
0.672	0.121	0.113	0.094
0.131	0.132	0.691	0.046
0.153	0.199	0.371	0.277
0.166	0.169	0.220	0.445
0.187	0.334	0.180	0.299
0.300	0.106	0.470	0.125

## Discussion

Dimerization is an inherent property of metazoan TFs and plays an important role in transcriptional regulation underlying differential gene expression. Multiple studies showed that dimerization of TFs can influence the proximity and the orientation of the implicated DNA binding domains, and as a consequence, it forces TF complexes to recognize a specific DNA site that is distinct from those recognized by the individual TFs (51–54). It has also been established that during the assembly of a heterodimer on DNA, the monomer-DNA intermediate tends to be kinetically less stable relative to the dimer-DNA complex (55–57). However, none of these studies provided to our knowledge a quantitative link between cooperative dimer-DNA interactions and the respective binding specificity model.

To interrogate the complex DNA binding behavior of heterodimers in a quantitative manner, we implemented in this study a novel integrative framework in which we coupled an in-depth biophysical on-chip characterization of PPARγ:RXRα binding to DNA with *in silico* modeling of the dimer-DNA association process. The highly parallel on-chip measurements thereby allowed us to simultaneously probe the binding of our focal proteins to multiple DNA sites under uniform conditions. This in turn allowed us to directly determine and compare the relative affinities of PPARγ, RXRα, and PPARγ:RXRα to various target sites that have previously been demonstrated to be of great functional importance (35, 38). These experiments revealed that RXRα binding is constrained to the AGGTCA hexamer such that even a single substitution within this site can cause a significant change in binding energy, consistent with data from previous studies (19, 58). Because of the sequence symmetry in PPRE, we found that RXRα can bind to either of the two hexameric half-sites (Fig. 3, *B* and *C*). In contrast, PPARγ alone did not have high affinity for PPRE *in vitro* (Fig. 3, *C* and *D*), but instead it exhibited high affinity for the PAL3 element (Fig. 3, *E* and *G*). Our results thereby suggest that PPARγ binds to PAL3 in monomeric rather than the previously proposed dimeric format (37), although further analyses will be required to formally validate this finding. These results raise the question as to why PPARγ is seldom associated with a PAL3 site *in vivo* (30) and why heterodimeric DNA binding by PPARγ and RXRα is preferred over the PPARγ-DNA or RXRα-DNA interactions. This question is especially relevant because the nuclear abundance of RXRα is much greater than that of PPARγ (47), which should theoretically favor the formation of RXRα-DNA complexes. Results from our analyses now indicate that the specificity of the heterodimer, even though somewhat dispersed among different response elements, is different from that identified for each partner independently (Fig. 2*B*). We also found that the extent of DNA binding of the heterodimer depends on the concentration of PPARγ and that the two TF partners contribute to the total binding energy of the interaction in a non-linear and non-additive fashion (Fig. 4, *A* and *B*). This significantly influences the shape of experimental binding curves such that it can no longer be explained with simple kinetic models (Fig. 4*A*), implying complex cooperative effects between the implicated factors and DNA that may promote heterodimer DNA binding.

To further dissect the nature of these cooperative interactions and to characterize the strength of cooperative heterodimer DNA binding with respect to the composition of the target site, we built a mechanistic model that accounts for all possible intermediate and final complexes that can occur between the three focal components. Mechanistic modeling so far has been widely applied in various studies to describe the kinetics of enzymatic and metabolic pathways (59–61) and even to characterize the *lac* operon function in *E. coli* (48). However, it has to our knowledge so far never been applied to comprehensively interpret high throughput heterodimer-DNA binding data. In contrast to the previously proposed quantitative models (62), the mechanistic approach did not require us to model the binding of a heterodimer to DNA as a one-step event nor to restrain the complex association to follow a monomer or a dimer pathway (55, 63). Rather, we aimed to account for the cooperative nature of these interactions and determine comprehensive binding parameters (Figs. 4*C*, 5*A,* and 6*A*). As such, we were able to determine the apparent affinity constant of the heterodimer that does not depend on the order of binding events, providing a novel framework to quantitatively interrogate heterodimer-DNA interactions (Fig. 4*C*, 6*A*). Importantly, this affinity constant does account for cooperative heterodimer-DNA binding, which, we showed, increases the *in vivo* DNA binding predictive power of our binding specificity model compared with a regular one-site equilibrium binding model.

Experimental MITOMI data further showed that the extent of cooperative effects in PPARγ:RXRα DNA binding depends on the orientation and nucleotide composition of the target site (Fig. 5*B*). Our model revealed that these patterns are associated more with PPARγ DNA binding rather than RXRα DNA interactions. Particularly, nucleotide alterations in the first part of the element resulted in greater variability of the cooperativity constant (as compared with the second part of PPRE) (Fig. 5*A*), which serves as the principal PPARγ:DNA binding interface (45). This observation implies that PPARγ plays an important role in mediating the specificity of the dimer as well as the strength of heterodimer DNA binding to a particular site.

It is thereby important to point out that our model does not elucidate the molecular origin of cooperativity as it does not distinguish between direct protein-protein interaction effects or indirect effects involving, for example, conformational state changes of implicated molecules (29). Nevertheless, the observed variability of the derived parameter ω as well as the *K_DoD_* constant reveals the versatile nature of heterodimer-DNA binding at single base pair resolution. This finding clearly suggests that we need to account for this variation when aiming to accurately model the PPARγ:RXRα-DNA interactions and to subsequently derive a comprehensive specificity matrix. Achieving such a robustness requires a comprehensive training set of input parameters however, which in turn demands a rigorous quantification of the focal molecular interactions (*i.e.* the binding of each dimer partner to DNA) prior to model simulation. This exposes an important limitation of the utilized mechanistic model in that it requires extensive quantitative binding data to accurately predict the DNA binding behavior of heterodimers. However, given the increasing availability of powerful assays such as MITOMI enabling the systematic analysis of protein-protein and protein-DNA interactions, we think that our modeling approach has great potential to further unravel the complex nature of protein-DNA interactions and go beyond the mere evaluation of binding strength. This may apply not only to heterodimers, but also to even higher order complexes involving allosteric interactions between TFs, co-factors, ligands, and DNA (64, 65). Nevertheless, despite our advance in deriving a DNA binding affinity constant of a heterodimer based on equilibrium-state measurements, our understanding of the kinetic mechanisms underlying the formation of heterodimers and their stabilization on DNA remains a challenging task. Follow-up studies may in this regard involve real time kinetic analyses of heterodimer-DNA complex formation for which the presented equilibrium binding data should prove highly valuable.

## Author Contributions

A. I. and B. D. designed the study and wrote the paper. A. I. performed the *in vitro* measurements. A. I., Y. B., and V. H. performed the mechanistic modeling.

## Supplementary Material

Supplemental Data

## Footnotes

4 The abbreviations used are:

TF: transcription factor
PPARγ: peroxisome proliferator-activated receptorγ
RXRα: retinoid X receptor α
PPRE: peroxisome proliferator response element
eGFP: enhanced GFP
MITOMI: mechanically induced trapping of molecular interactions.
