# Supplementary material for: Quantification of Cooperativity in Heterodimer-DNA Binding Improves the Accuracy of Binding Specificity Models
Source: J Biol Chem. 2016 Feb 24;291(19):10293–306. doi: 10.1074/jbc.M115.691154 (PMC4858977; doi:10.1074/jbc.M115.691154)
Supplement: Supplemental Data [file supp_291_19_10293__index.html]

Quantification of cooperativity in heterodimer-DNA binding improves the accuracy of binding specificity models — Quantification of Cooperativity in Heterodimer-DNA Binding Improves the Accuracy of Binding Specificity Models — Quantification of Cooperativity in Heterodimer-DNA Binding — Supplemental Data 

# Quantification of Cooperativity in Heterodimer-DNA Binding Improves the Accuracy of Binding Specificity Models

## Supplemental Data

- Supplementary data (.pdf, 3.7 MB) - Supplementary data to be published online.
