# Supplementary material for: Quantification of Cooperativity in Heterodimer-DNA Binding Improves the Accuracy of Binding Specificity Models
Source: J Biol Chem. 2016 Feb 24;291(19):10293–306. doi: 10.1074/jbc.M115.691154 (PMC4858977; doi:10.1074/jbc.M115.691154)
Supplement: Supplemental Data [file 10.1074_M115.691154_jbc.M115.691154-1.pdf]

Extended experimental and modeling data

Table 1. Model input parameters, K_{DoD} and cooperativity parameters of PPARg:RXRa heterodimer binding to PPRE as estimated by the cooperativity model

PPRE Sequence	K_00,10 (=K_1,0) M-1	σ (K_00,10) M-1	K_00,20 (=K_2,0) M-1	σ (K_00,20) M-1	ω_1,2	ω_2,2	K_DoD M-2	RSS
AAACTAGGTCAAAGGTCAAA	2.08E+06	1.20E+05	1.98E+08	1.01E+07	3.56E+07	8.04E+07	1.46E+22	0.084
AAACTAGGTCAAAGGTCATA	1.23E+06	1.32E+05	3.78E+07	4.35E+06	2.23E+03	2.06E+04	1.04E+17	0.271
AAACTAGGTCAAAGGTCACA	2.33E+06	1.34E+05	1.22E+08	3.19E+07	4.59E+06	4.77E+06	1.31E+21	0.434
AAACTAGGTCAAAGGTCAGA	1.53E+06	7.23E+04	1.10E+07	2.10E+06	1.33E+06	1.08E+07	2.23E+19	0.656
AAACTAGGTCAAAGGTCAAT	2.38E+06	1.64E+05	2.79E+08	6.64E+07	3.80E+06	1.40E+05	2.52E+21	0.162
AAACTAGGTCAAAGGTCAAC	2.55E+06	2.78E+05	9.97E+08	3.88E+07	3.94E+05	1.50E+03	1.00E+21	0.261
AAACTAGGTCAAAGGTCAAG	1.65E+06	4.16E+04	1.00E+09	1.10E+08	1.01E+09	9.38E+06	1.67E+24	0.229
TAACTAGGTCAAAGGTCAAA	8.46E+06	2.43E+05	1.01E+08	1.91E+07	1.76E+06	1.17E+07	1.51E+21	0.172
CAACTAGGTCAAAGGTCAAA	1.21E+06	9.25E+04	1.17E+08	1.97E+07	2.80E+03	9.94E+03	3.95E+17	0.192
GAACTAGGTCAAAGGTCAAA	1.19E+06	1.84E+05	1.00E+08	1.16E+07	3.28E+03	1.57E+04	3.92E+17	0.183
ATACTAGGTCAAAGGTCAAA	5.83E+05	5.69E+04	1.45E+08	4.33E+07	6.04E+07	1.07E+08	5.12E+21	0.246
ACACTAGGTCAAAGGTCAAA	7.23E+05	4.34E+04	4.33E+07	4.92E+06	1.20E+04	5.95E+04	3.77E+17	0.042
AGACTAGGTCAAAGGTCAAA	8.27E+05	4.64E+04	2.05E+08	1.38E+07	2.03E+06	2.91E+06	3.45E+20	0.042
AATCTAGGTCAAAGGTCAAA	2.75E+06	2.28E+05	1.98E+08	5.25E+07	7.01E+07	1.52E+08	3.81E+22	0.097
AACCTAGGTCAAAGGTCAAA	2.86E+05	2.28E+05	1.25E+08	7.54E+07	7.10E+06	2.96E+06	2.53E+20	0.009
AAGCTAGGTCAAAGGTCAAA	2.46E+05	5.72E+03	5.27E+07	6.39E+06	3.26E+05	1.74E+05	4.22E+18	0.029
AAAATAGGTCAAAGGTCAAA	1.05E+06	1.06E+05	1.64E+08	1.52E+07	4.03E+03	6.25E+03	6.96E+17	0.040
AAATTAGGTCAAAGGTCAAA	1.70E+06	3.73E+05	2.59E+08	1.86E+07	7.34E+06	5.51E+06	3.22E+21	0.040
AAAGTAGGTCAAAGGTCAAA	3.52E+06	3.05E+05	1.38E+08	2.54E+07	1.61E+06	9.98E+06	7.84E+20	0.066
AAACAAGGTCAAAGGTCAAA	3.60E+05	2.76E+04	7.87E+07	2.41E+07	2.29E+08	2.13E+08	6.48E+21	0.032
AAACCAGGTCAAAGGTCAAA	3.82E+05	5.64E+04	3.82E+08	1.29E+07	2.13E+02	2.66E+02	3.12E+16	0.095
AAACGAGGTCAAAGGTCAAA	2.96E+05	8.56E+04	9.82E+07	7.68E+06	6.98E+03	1.83E+04	2.03E+17	0.062
AAACTTGGTCAAAGGTCAAA	1.16E+05	6.46E+03	4.68E+06	2.16E+05	3.58E+06	3.21E+07	1.95E+18	0.038
AAACTCGGTCAAAGGTCAAA	2.15E+05	5.91E+03	8.80E+06	7.57E+05	1.49E+06	1.04E+07	2.82E+18	0.096
AAACTGGGTCAAAGGTCAAA	2.27E+06	1.84E+05	1.73E+08	1.30E+07	1.40E+08	4.79E+08	5.48E+22	0.282
AAACTAAGTCAAAGGTCAAA	1.59E+05	5.62E+04	7.36E+06	9.64E+05	2.05E+08	1.26E+09	2.40E+20	0.064
AAACTACGTCAAAGGTCAAA	9.44E+04	3.32E+04	6.66E+06	1.54E+06	2.07E+05	3.43E+05	1.30E+17	0.037
AAACTATGTCAAAGGTCAAA	2.68E+05	1.26E+04	9.75E+06	6.34E+05	5.57E+08	1.86E+09	1.46E+21	0.083
AAACTAGATCAAAGGTCAAA	1.84E+05	4.90E+04	4.91E+06	4.72E+05	1.62E+08	1.10E+09	1.46E+20	0.111
AAACTAGCTCAAAGGTCAAA	1.06E+05	5.47E+04	9.80E+05	2.83E+04	2.04E+09	3.62E+10	2.13E+20	0.034
AAACTAGTTCAAAGGTCAAA	1.23E+05	3.22E+04	2.54E+07	3.83E+06	7.18E+04	6.85E+04	2.25E+17	0.134
AAACTAGGACAAAGGTCAAA	3.79E+05	2.29E+04	2.04E+06	1.41E+05	1.03E+09	9.14E+09	7.92E+20	0.086
AAACTAGGCCAAAGGTCAAA	6.22E+05	6.69E+04	5.86E+06	6.23E+05	1.21E+10	4.02E+11	4.41E+22	0.243
AAACTAGGGCAAAGGTCAAA	6.86E+05	1.02E+05	9.19E+06	8.21E+05	1.51E+09	1.13E+10	9.54E+21	0.018
AAACTAGGTAAAAGGTCAAA	5.15E+05	3.40E+04	3.42E+06	2.18E+05	2.01E+05	1.11E+07	3.55E+17	0.204

AAACTAGGTTAAAGGTCAAA	7.16E+05	9.70E+05	1.70E+07	1.34E+06	1.39E+04	7.87E+04	1.69E+17	0.156
AAACTAGGTGAAAGGTCAAA	2.23E+06	1.12E+05	2.66E+07	5.23E+06	3.12E+02	1.08E+03	1.85E+16	0.149
AAACTAGGTCTAAGGTCAAA	1.74E+05	4.81E+04	8.02E+05	2.72E+04	5.68E+06	5.28E+07	7.91E+17	0.009
AAACTAGGTCCAAGGTCAAA	1.44E+05	3.15E+04	3.68E+05	2.58E+04	2.40E+11	1.15E+13	1.27E+22	0.063
AAACTAGGTCGAAGGTCAAA	3.94E+05	7.31E+04	5.54E+06	1.42E+05	5.41E+05	1.50E+06	1.18E+18	0.589
AAACTAGGTCACAGGTCAAA	1.67E+06	6.23E+05	7.95E+06	4.61E+05	1.53E+04	2.94E+05	2.02E+17	0.362
AAACTAGGTCATAGGTCAAA	1.06E+06	2.59E+05	4.38E+07	5.05E+06	8.61E+06	9.90E+06	3.98E+20	0.105
AAACTAGGTCAGAGGTCAAA	1.27E+06	1.13E+05	2.69E+07	2.69E+06	3.40E+06	1.13E+07	1.16E+20	0.271
AAACTAGGTCAATGGTCAAA	7.91E+05	1.24E+05	3.98E+05	3.08E+04	1.15E+05	3.26E+07	3.63E+16	0.381
AAACTAGGTCAACGGTCAAA	6.16E+05	1.61E+05	1.05E+06	3.99E+04	2.93E+05	9.40E+07	1.90E+17	0.293
AAACTAGGTCAAGGGTCAAA	8.38E+05	2.25E+05	1.61E+07	1.68E+06	8.73E+03	5.58E+04	1.18E+17	0.311
AAACTAGGTCAAACGTCAAA	4.86E+05	1.44E+05	8.60E+05	4.27E+04	6.65E+05	1.34E+08	2.78E+17	0.062
AAACTAGGTCAAAAGTCAAA	4.99E+05	1.15E+05	2.41E+06	1.70E+05	2.19E+05	7.83E+06	2.63E+17	0.111
AAACTAGGTCAAATGTCAAA	1.32E+06	2.81E+05	5.14E+06	3.14E+05	4.26E+01	8.63E+02	2.88E+14	0.117
AAACTAGGTCAAAGCTCAAA	4.51E+05	1.34E+05	2.18E+05	5.69E+03	2.44E+09	1.47E+10	2.40E+20	0.026
AAACTAGGTCAAAGATCAAA	5.93E+05	3.42E+04	2.73E+06	1.58E+05	2.61E+07	1.86E+07	4.23E+19	0.047
AAACTAGGTCAAAGTTCAAA	1.57E+06	6.33E+04	4.44E+07	2.30E+06	3.08E+07	1.01E+06	2.14E+21	0.757
AAACTAGGTCAAAGGACAAA	9.98E+05	8.48E+04	9.06E+05	3.85E+04	2.45E+07	4.33E+07	2.22E+19	0.398
AAACTAGGTCAAAGGCCAAA	6.04E+05	1.42E+05	3.59E+06	2.31E+05	1.15E+07	7.53E+05	2.50E+19	0.393
AAACTAGGTCAAAGGGCAAA	1.57E+06	1.20E+05	1.33E+07	8.40E+05	6.73E+06	7.41E+05	1.40E+20	0.171
AAACTAGGTCAAAGGTGAAA	3.08E+06	7.80E+04	1.32E+07	5.04E+05	6.25E+06	8.50E+05	2.55E+20	0.130
AAACTAGGTCAAAGGTAAAA	1.09E+06	9.83E+04	4.39E+06	3.43E+05	6.82E+07	1.70E+08	3.28E+20	0.131
AAACTAGGTCAAAGGTTAAA	2.12E+06	1.59E+05	1.79E+07	2.14E+06	1.85E+08	7.64E+07	7.05E+21	0.248
AAACTAGGTCAAAGGTCTAA	8.43E+05	1.23E+05	4.55E+06	2.34E+05	1.20E+07	5.38E+07	4.61E+19	0.164
AAACTAGGTCAAAGGTCCAA	7.31E+05	3.54E+04	1.56E+06	8.43E+04	1.39E+07	5.22E+07	1.58E+19	0.445
AAACTAGGTCAAAGGTCGAA	1.55E+06	1.13E+05	2.70E+08	8.29E+07	6.63E+08	4.54E+07	2.79E+23	0.571

PPRE Sequence: Mutation library of the PPRE element

K_00,10: binding affinity of PPARg to PPRE

σ (K_00,10) M-1 : standard deviation of the K_00,10

K_00,20: binding affinity of RxRa to PPRE

σ (K_00,20) M-1 : standard deviation of the K_00,20

K_D1: PPARg-RxRa proteins affinity

K_D2: RxRa-RxRa proteins affinity

ω_1,2: PPARg-RxRa cooperativity

ω_2,2: RxRa-RxRa cooperativity
RSS: the residual sum of squares value of the cooperativity model fits
The effect of the mutations on the binding affinity has been independently measured for the two proteins and inserted in the model as input parameter.

K_D1 ± σ, M-1	K_D2 ± σ, M-1
(6.13 ± 0.13) E+07	(5.13±0.09) E+07

Figure S1. Model fits of RXRα-PPRE experimental binding curves. The mechanistic model is streamlined for monomeric TF-DNA binding in this case and thus restrained to consider RXRα, PPRE, RXRα-PPRE interactions. Residuals, calculated for each

sequence, are represented on each plot above the respective sequence fit and are randomly scattered around zero, indicating an accurate model fit.

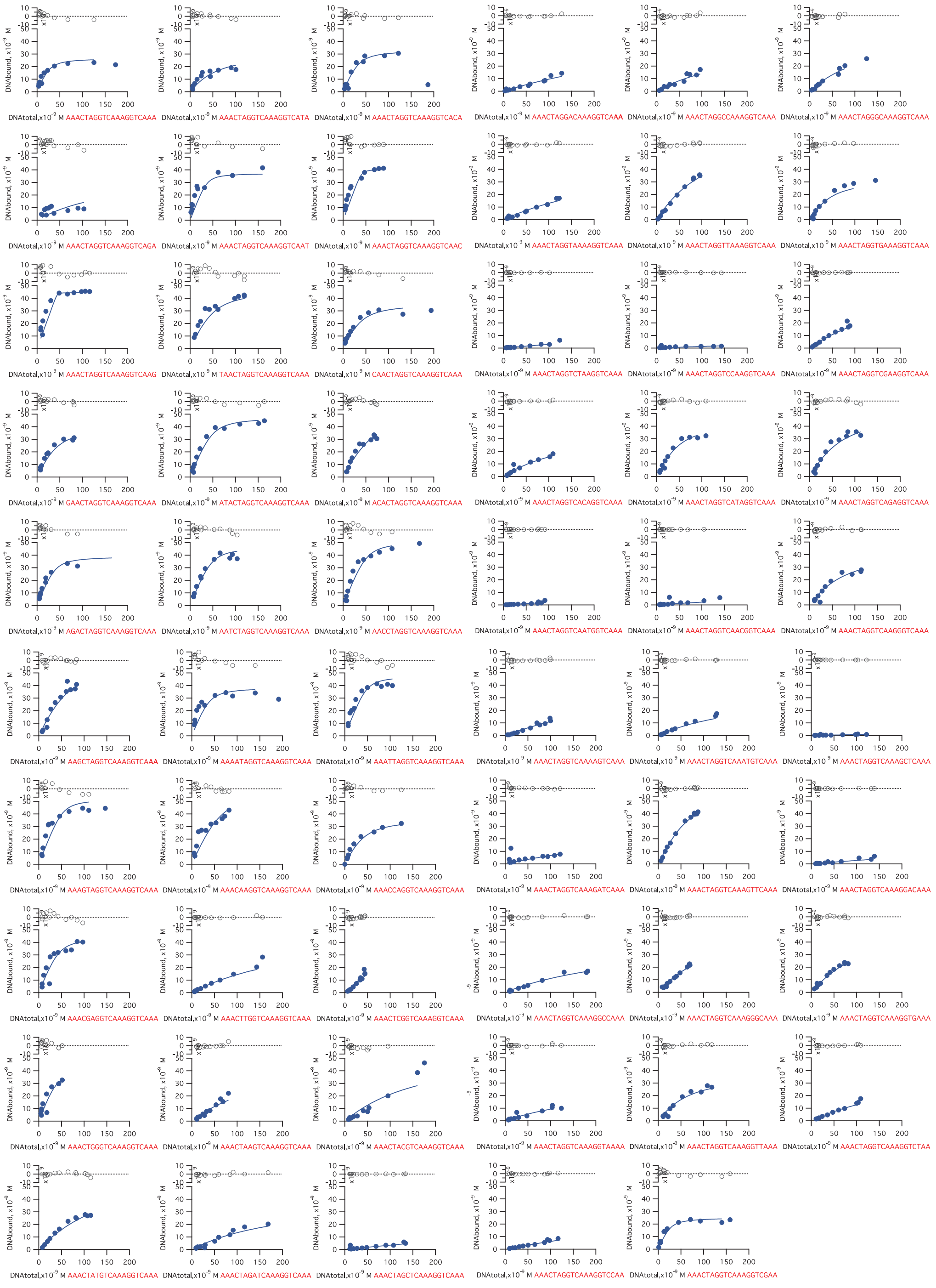

FigureS2. Same as **S1** but for PPARγ.

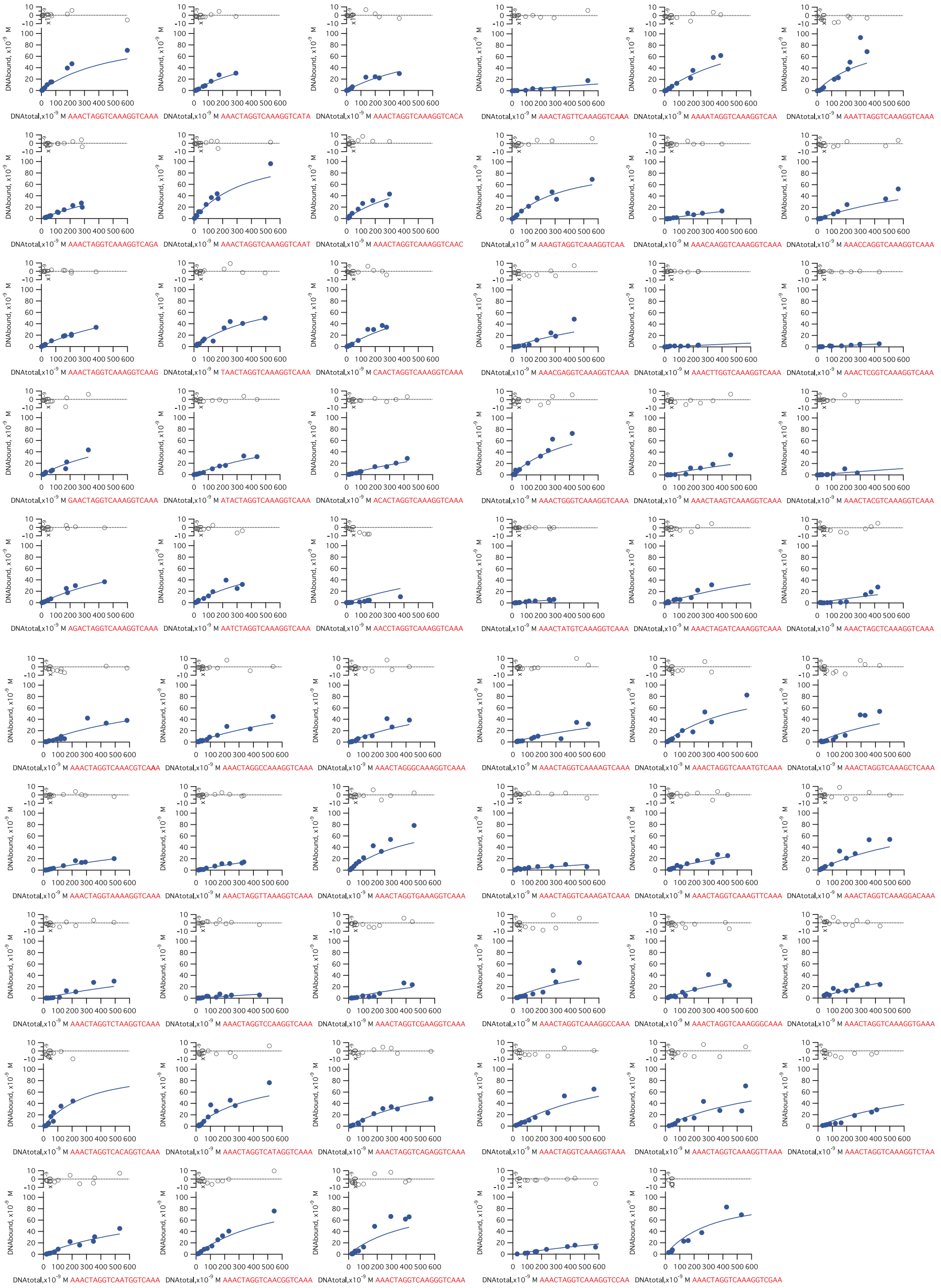

FigureS3. Performance of the mechanistic model solved for equilibrium. Examples of experimental data corresponding to each tested PPRE variant and corresponding binding curves as predicted by the model when ether accounting for cooperativity (red curves) or not (green curves). The sum of squared residuals of both cooperativity and “no cooperativity” model fits are indicated for each PPRE mutant (in red and green respectively) and plotted for each sequence as a bar plot at the bottom of the figure for a direct comparison.

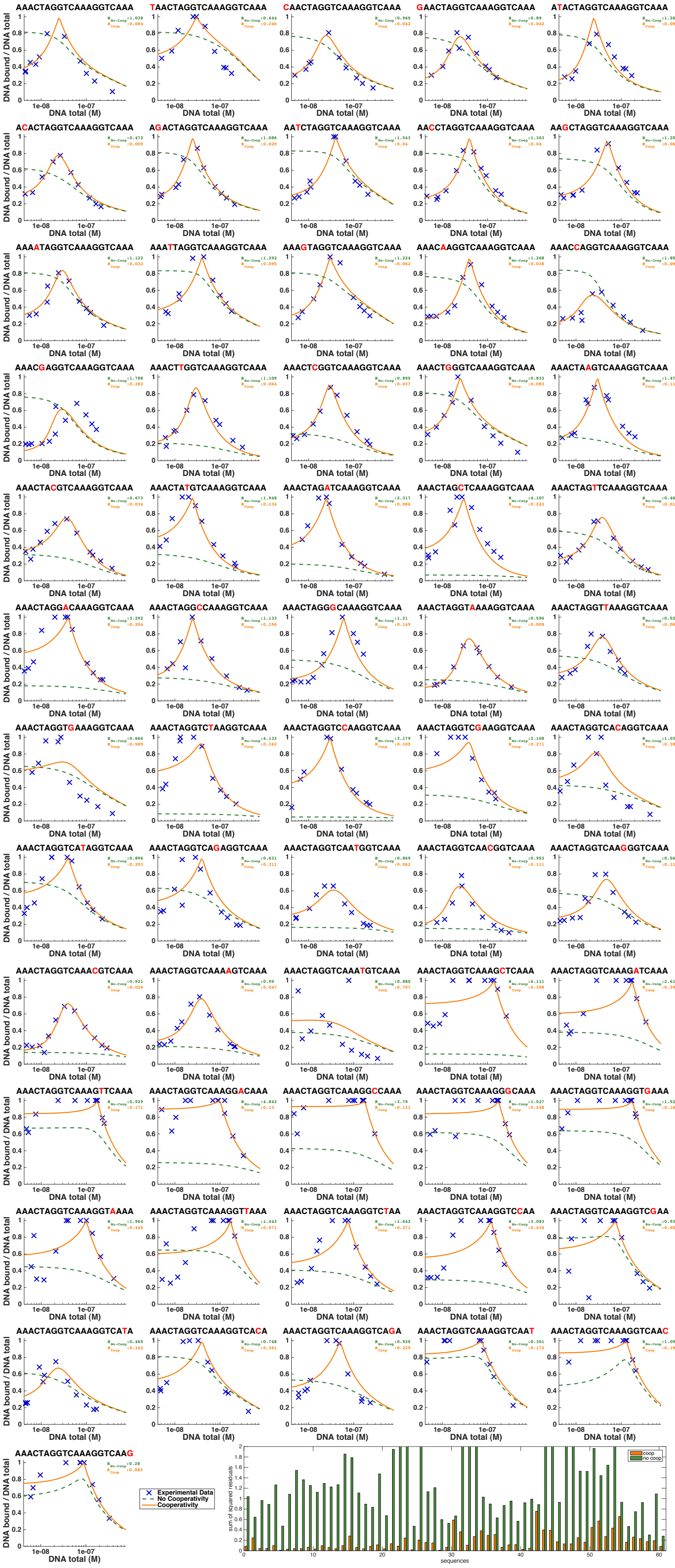
AAACTAGGTCAAAGGTCAAA
TAACTAGGTCAAAGGTCAAA
CAACTAGGTCAAAGGTCAAA
GAACTAGGTCAAAGGTCAAA
ATACTAGGTCAAAGGTCAAA
ACACTAGGTCAAAGGTCAAA
AGACTAGGTCAAAGGTCAAA
AATCTAGGTCAAAGGTCAAA
AACCTAGGTCAAAGGTCAAA
AAGCTAGGTCAAAGGTCAAA
AAAATAGGTCAAAGGTCAAA
AAATTAGGTCAAAGGTCAAA
AAAGTAGGTCAAAGGTCAAA
AAACAAGGTCAAAGGTCAAA
AAACCAGGTCAAAGGTCAAA
AAACGAGGTCAAAGGTCAAA
AAACTTGGTCAAAGGTCAAA
AAACTCGGTCAAAGGTCAAA
AAACTGGGTCAAAGGTCAAA
AAACTAAGTCAAAGGTCAAA
AAACTACGTCAAAGGTCAAA
AAACTATGTCAAAGGTCAAA
AAACTAGATCAAAGGTCAAA
AAACTAGCTCAAAGGTCAAA
AAACTAGTTCAAAGGTCAAA
AAACTAGGACAAAGGTCAAA
AAACTAGGCCAAAGGTCAAA
AAACTAGGGCAAAGGTCAAA
AAACTAGGTAAAAGGTCAAA
AAACTAGGTTAAAGGTCAAA
AAACTAGGTGAAAGGTCAAA
AAACTAGGTCTAAGGTCAAA
AAACTAGGTCCAAGGTCAAA
AAACTAGGTCGAAGGTCAAA
AAACTAGGTCACAGGTCAAA
AAACTAGGTCATAGGTCAAA
AAACTAGGTCAGAGGTCAAA
AAACTAGGTCAATGGTCAAA
AAACTAGGTCAACGGTCAAA
AAACTAGGTCAAGGGTCAAA
AAACTAGGTCAAACGTCAAA
AAACTAGGTCAAAAGTCAAA
AAACTAGGTCAAATGTCAAA
AAACTAGGTCAAAGCTCAAA
AAACTAGGTCAAAGATCAAA
AAACTAGGTCAAAGTTCAAA
AAACTAGGTCAAAGGACAAA
AAACTAGGTCAAAGGCCAAA
AAACTAGGTCAAAGGGCAAA
AAACTAGGTCAAAGGTGAAA
AAACTAGGTCAAAGGTAAAA
AAACTAGGTCAAAGGTTAAA
AAACTAGGTCAAAGGTCTAA
AAACTAGGTCAAAGGTCCAA
AAACTAGGTCAAAGGTCGAA
AAACTAGGTCAAAGGTCATA
AAACTAGGTCAAAGGTCACA
AAACTAGGTCAAAGGTCAGA
AAACTAGGTCAAAGGTCAAT
AAACTAGGTCAAAGGTCAAC
AAACTAGGTCAAAGGTCAAG
DNA bound / DNA total
DNA total (M)
1e-08
1e-07
Experimental Data
No Cooperativity
Cooperativity
sum of squared residuals
sequences
coop
no coop
